# IT’S A COOL LITTLE TOOL: PROVIDER PERSPECTIVES ON IMPLEMENTING PAL CARDS DURING COVID-19

**DOI:** 10.1093/geroni/igac059.439

**Published:** 2022-12-20

**Authors:** Katherine Abbott, Natalie Douglas

## Abstract

This symposium describes the implementation of a person-centered care (PCC) communication tool in nursing homes. PCC is a philosophy that recognizes “knowing the person” and honoring individual preferences. The communication tool is based on an assessment of NH resident likes and dislikes via the Preferences for Everyday Living Inventory (PELI). The PELI is an evidenced-based, validated instrument that can be used to enhance the delivery of PCC. The Preferences for Activity and Leisure (PAL) Card was developed to communicate important resident preferences across care team members. From July 2020 to July 2021 we lead a Quality Improvement Project (QIP) approved by the Ohio Department of Aging providing virtual coaching to providers who created 15-20 PAL Cards for their residents. Our first presentation describes the QIP where n=16 started implementation and n=11 communities completed the project during the height of the pandemic. Providers were non-profit (50%) with an average star rating of 3.29 (SD 1.33). Feedback from n=68 direct care staff on PAL Card usage are reported. The next four presentations describe implementation of PAL Cards from the perspective of the provider champions who contributed to n=66 monthly interviews. Interviews were recorded, transcribed verbatim, and coded in Dedoose using the Consolidated Framework for Implementation Research as an a priori coding scheme. We present barriers and facilitators related to the domains of Inner Setting, Characteristics of the Individual, Characteristics of the Intervention, and Process. The Discussant, Dr. Natalie Douglas will discuss the implications of initiatives to address the quality of resident care.

